# Transactions Texas State Medical Association, ’87

**Published:** 1887-08

**Authors:** 


### TRANSACTIONS TEXAS STATE MED. ASSOCIATION FOR 1887.

The volume of Transactions of the Austin meeting of the Texas State Medical Association is about finished, and will be mailed to members and exchanges by or before the 20th August, inst. It makes about 425 pages, and is bound in paper—for reasons that none can appreciate, except the Publishing Committee. It was printed by Von Boeckmann, of Austin, at $1 per page of 464 words, long primer type—the cheapest job ever turned out, considering the quality of paper and work. A review of its contents will appear in our next. We take occasion here to say, that those members who have not paid their dues for three years or more, and whose names have been dropped from the roll, need not feel disappointed if they fail to receive the volume; nor must they blame the Secretary, who is but doing a bounden duty.

